# Supplementary material for: Uncalled4 improves nanopore DNA and RNA modification detection via fast and accurate signal alignment
Source: Nat Methods. 2025 Mar 28;22(4):681–91. doi: 10.1038/s41592-025-02631-4 (PMC11978507; doi:10.1038/s41592-025-02631-4)
Supplement: Supplementary file 1 — Supplementary figures and notes. [file 41592_2025_2631_MOESM1_ESM.pdf]

nature methods

Supplementary information

https://doi.org/10.1038/s41592-025-02631-4

Uncalled4 improves nanopore DNA and RNA modification detection via fast and accurate signal alignment

In the format provided by the authors and unedited

Supplemental Figures

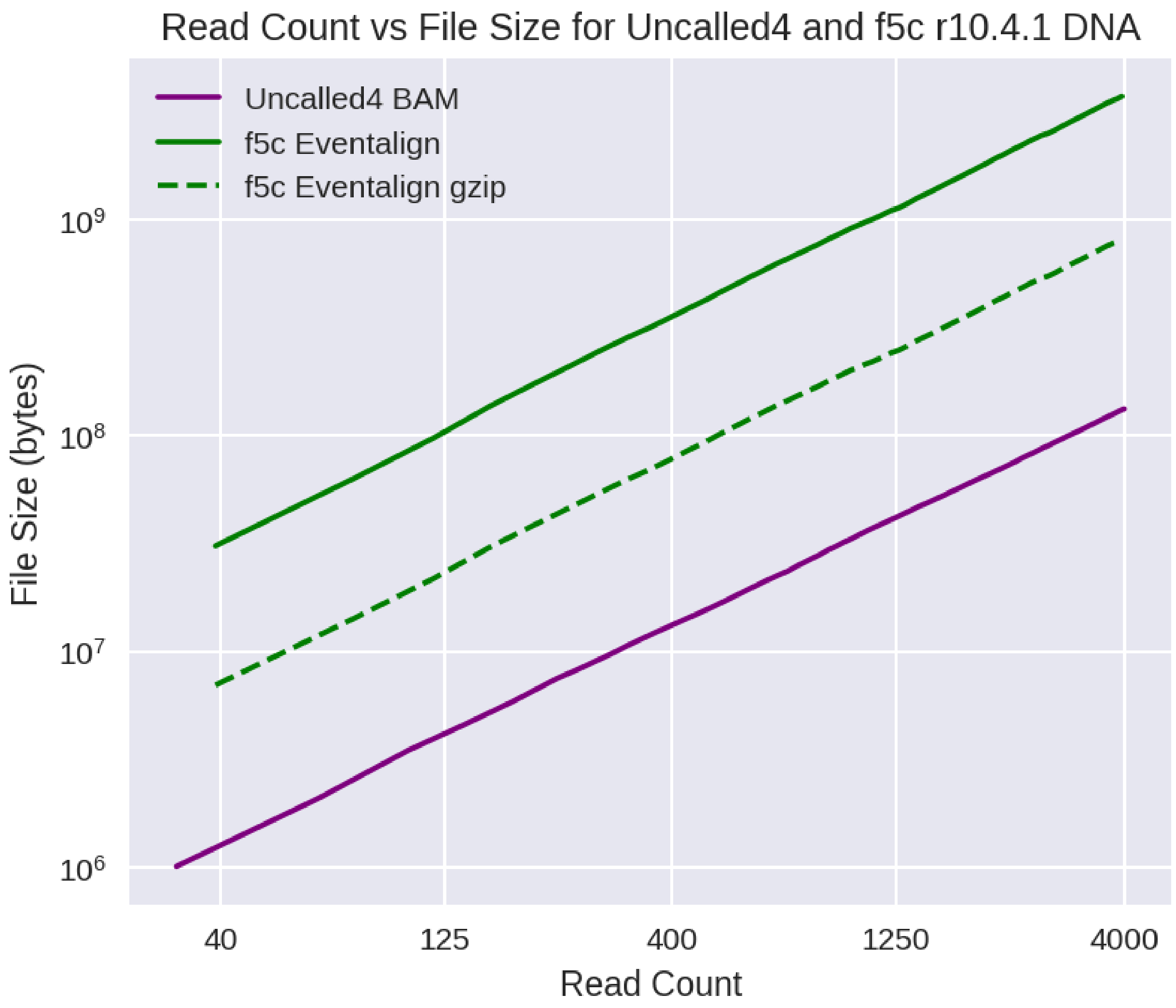

Supplemental Figure 1. File sizes of r10.4.1 DNA signal alignments using Uncalled4's BAM format for f5c eventalign format (also used by Nanopolish) with or without gzip compression. Both x- and y-axes are log scale.

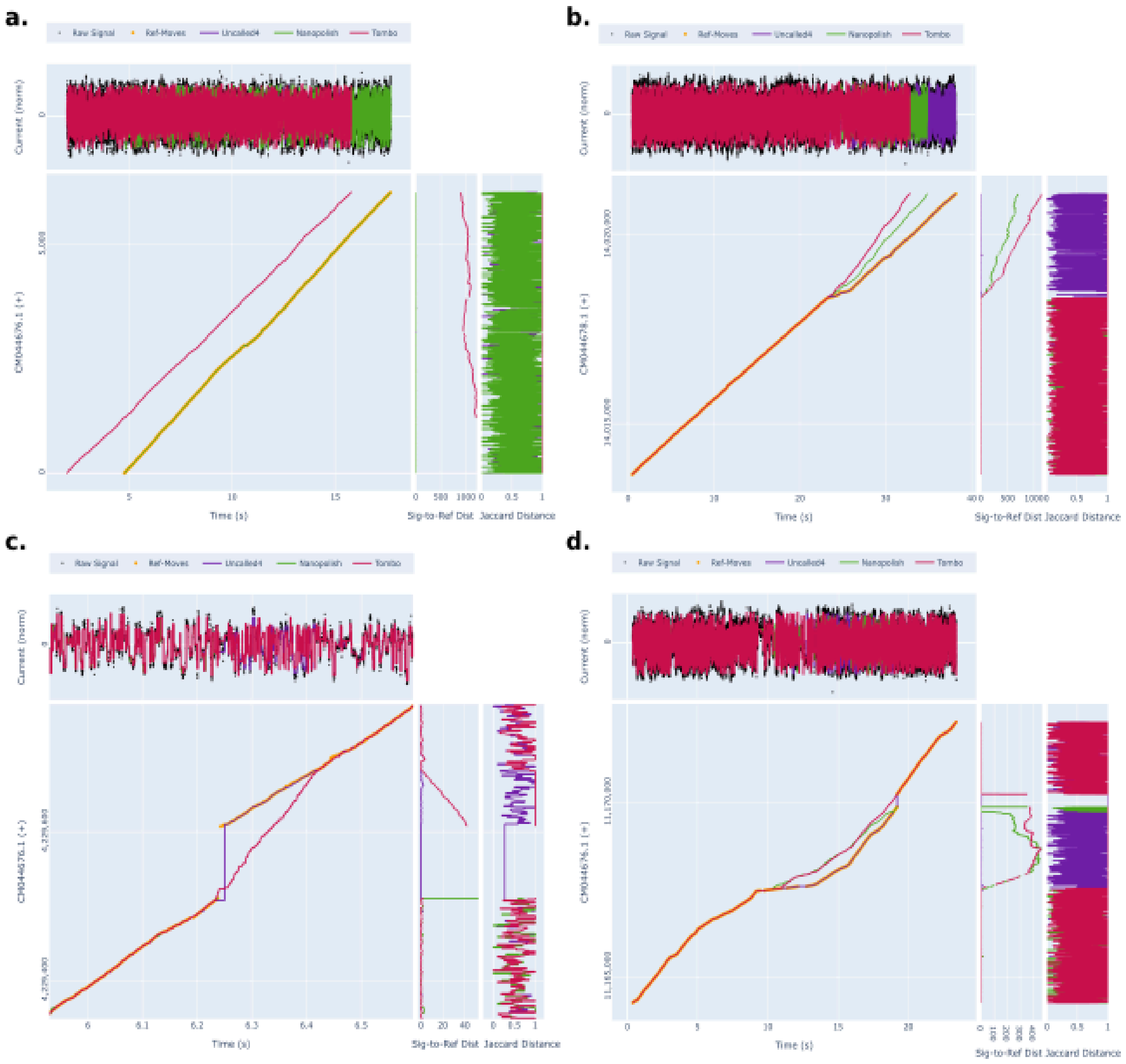

Supplemental Figure 2. Large-scale alignment errors in *D. melanogaster* DNA sequenced with r9.4.1. **(a)** An intergenic repeat causing Tombo to be shifted by several hundred bases. **(b)** A read which appears to change speed at the end, causing all three signal aligners to output different alignment endpoints, with Uncalled4 remaining close to the basecaller *ref-moves.* **(c)** A large deletion which Tombo aligns over, while Nanopolish and Uncalled4 properly skip the region. **(d)** A read with inconsistent speed, causing internal disruptions in Tombo and Nanopolish alignment.

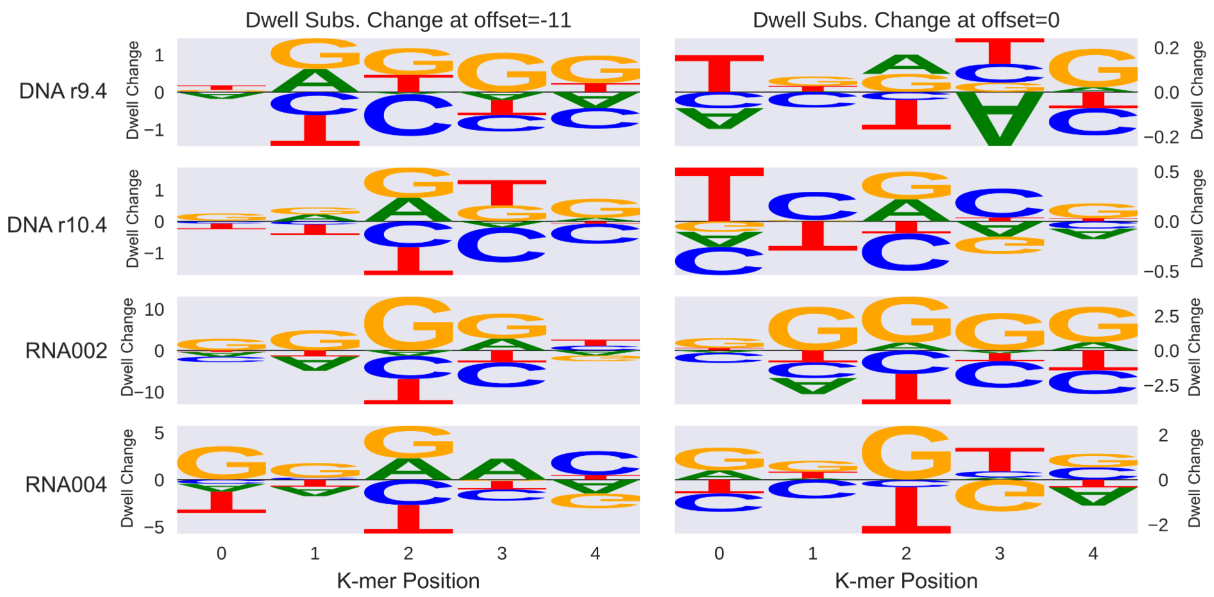

Supplemental Figure 3. Effect of 5-mer nucleotides on dwell time centered at 11 bases upstream the pore at the pore central position. Nucleotide heights indicate the mean change in dwell time observed when that base is substituted from another at each position, similar to the substitution matrices (**Fig. 2a**) but measuring relative dwell time rather than absolute current change. All plots use 5-mers since that is the smallest k-mer length of any pore model (RNA002).

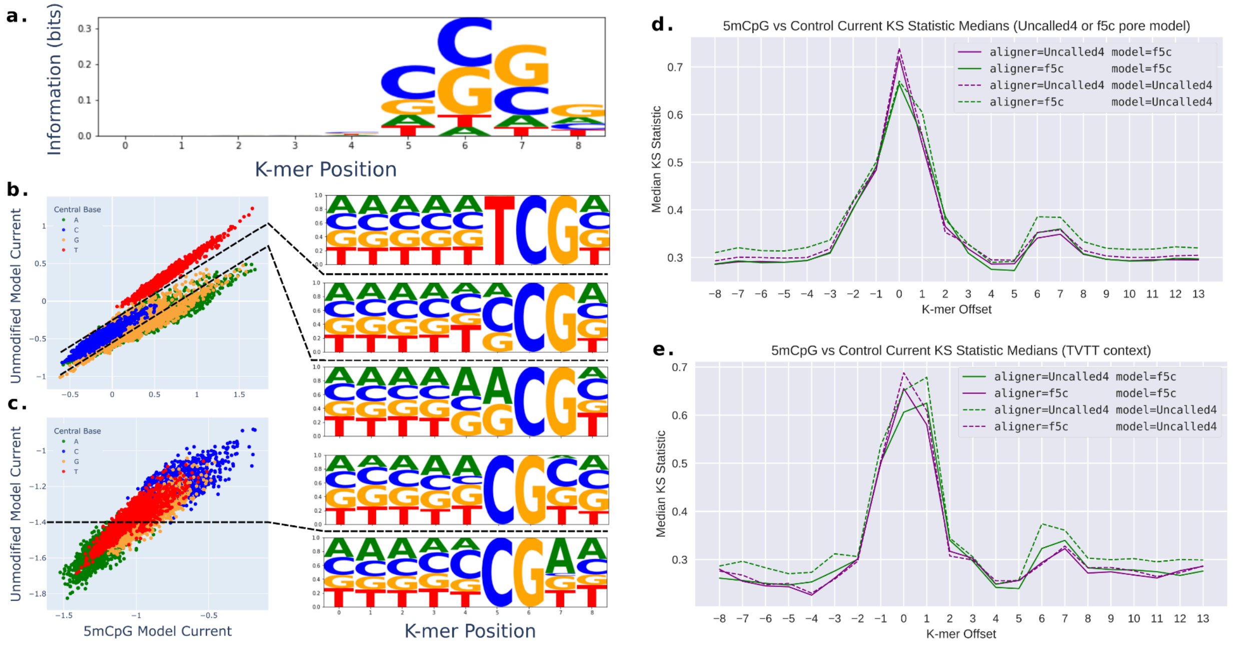

Supplemental Figure 4. 5mCpG model analysis. For comparison, the CpG model currents were linearly scaled using linear regression between non-CpG k-mers in Uncalled4's unmodified and 5mCpG models. **(a)** Per-base information content of k-mers with higher than median deviation between unmodified and 5mCpG models, showing CpG in any of the last four positions provides the most information. **(b)** Unmodified and 5mCpG currents for k-mers with CG in 6th and 7th positions, colored by the identity of the 5th position, alongside sequence logos for three subsets of k-mers divided by dashed lines. **(c)** Similar to (b) but for k-mer with CG in the 5th and 6th position, colored by 6th position identity and sequence logos for two subsets. **(d)** Current-level KS statistics of differences in current between control and 5mCpG using the Uncalled4-trained r10.4 pore model or the builtin f5c model. **(d)** The same KS statistics but filtered for only CpG sites which contain the TVTT motif within the 9-mer.

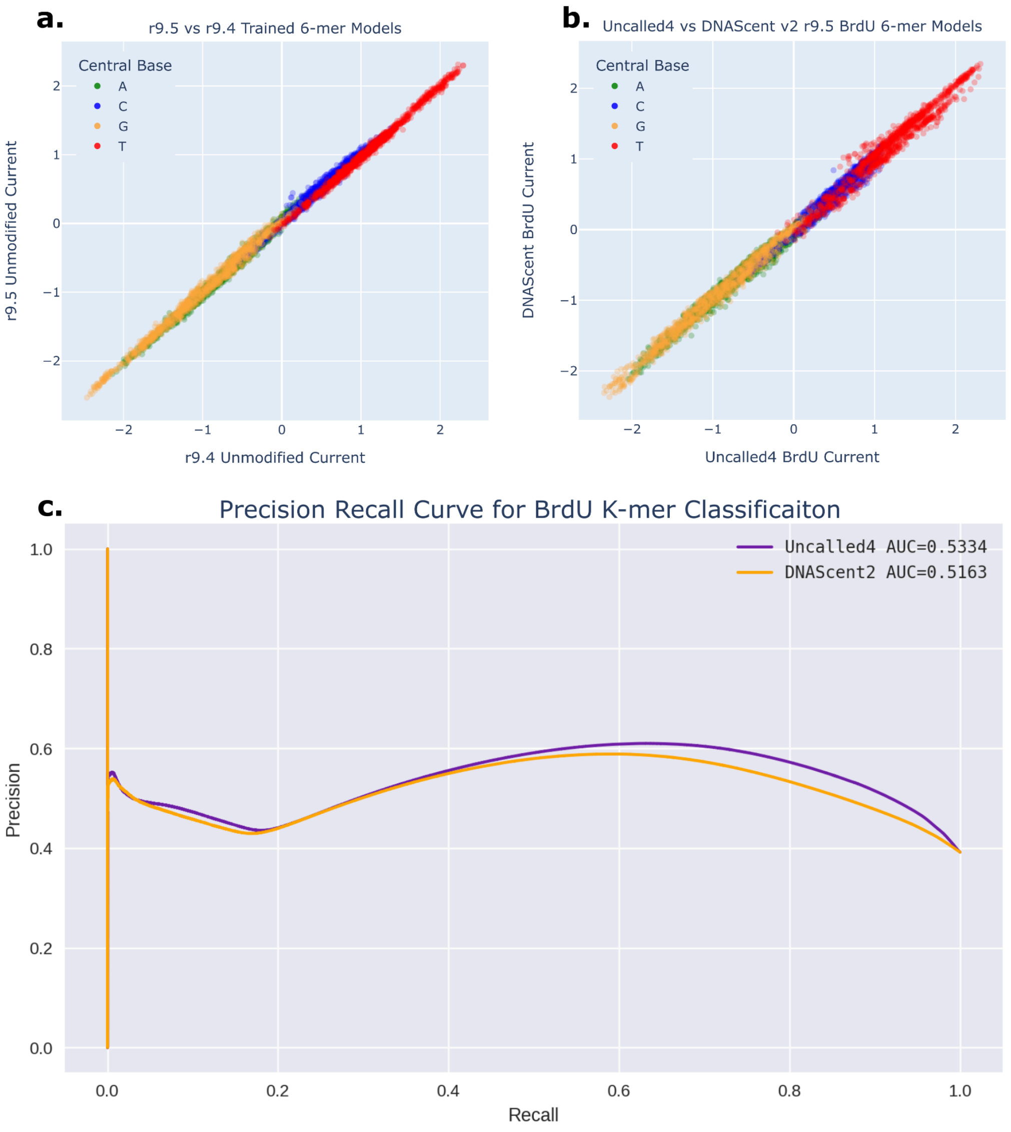

Supplemental Figure 5. BrdU model training and classification. **(a)** An Uncalled4 r9.5 6-mer model trained from unmodified *S. cerevisiae* DNA, compared to Uncalled4 trained r9.4 model. r9.5 was ONT's experimental "1D^2" sequencing chemistry, used the same pore as r9.4 with an alternate library preparation, resulting in a nearly identical k-mer model. **(b)** Comparison between Uncalled4 r9.5 BrdU model and the published DNAScent2 model. The DNAScent2 model only included k-mers which contained a "T", so missing k-mers were filled in using the Nanopolish model with which DNAScent was initialized. (**c**) Precision-recall curve showing classification of BrdU k-mers, generated by combining reads from the unmodified and 100% BrdU datasets, including k-mers with no "T"s and a single "T" in the center. Reads were aligned with Uncalled4 using either BrdU model or control, and the absolute difference in current between the BrdU and control models were used as the score for the precision-recall analysis.

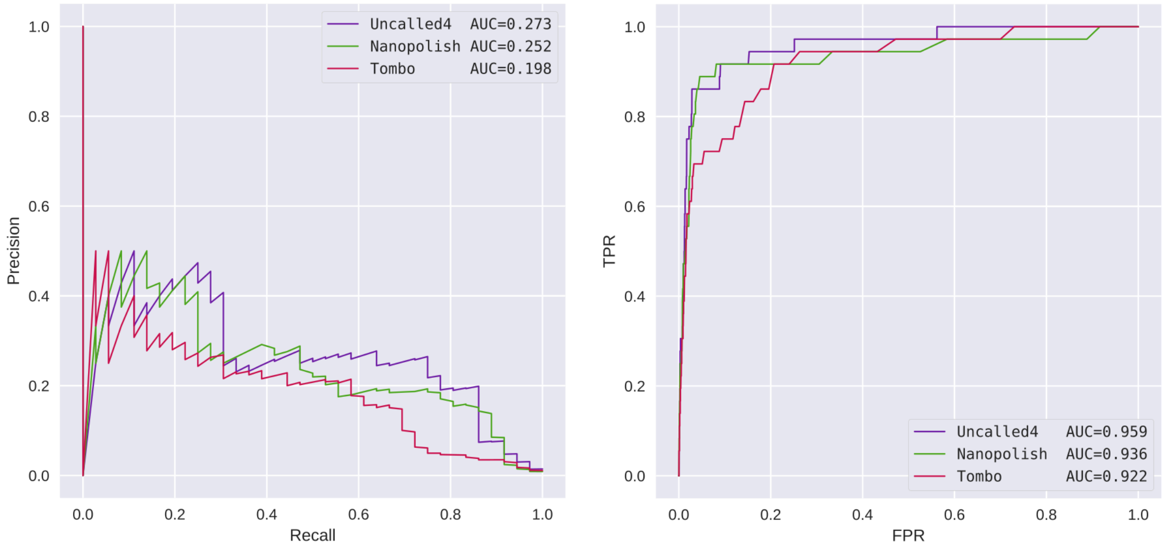

Supplemental Figure 6. Precision recall (left) and ROC (right) curves for detecting 36 modifications in *E. coli* ribosomal RNA, using KS statistics comparing current levels from native in *in vitro* transcribed RNA.

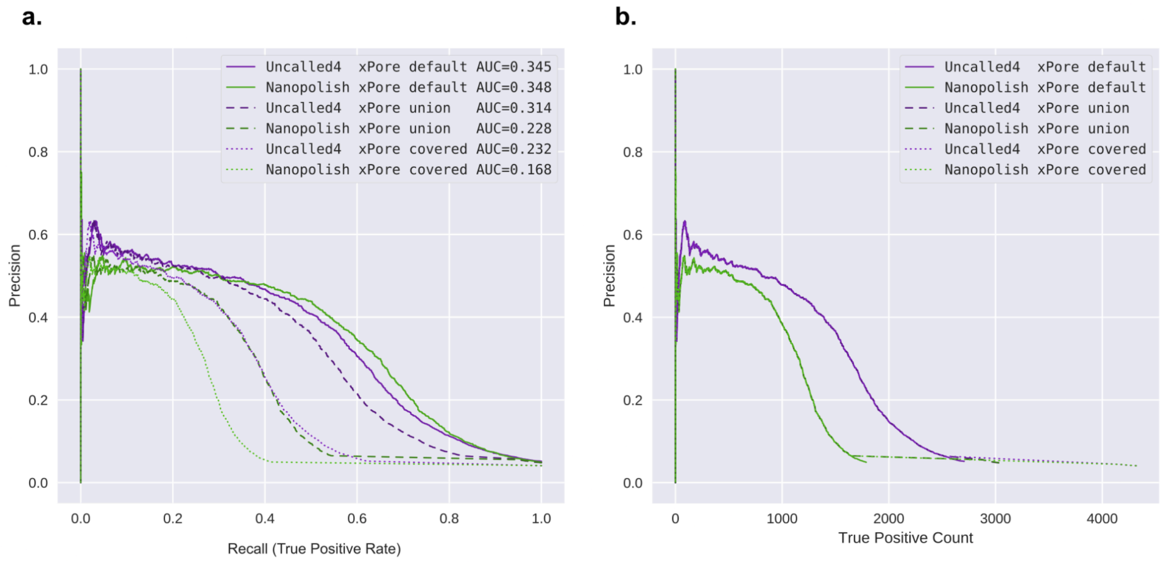

Supplemental Figure 7. (a) Precision recall curves for transcript-level m6A detection in HEK293t with xPore results, using three different methods for handling sites not output by xPore: "default" is naive comparison where missing values are ignored and the output sites and directly input to the precision/recall curve; "union" takes the union of all sites output by each aligner, and assigns a score of "0" for sites not output by either aligner, yielding a lower apparent recall for Nanopolish due to more missing data; "covered" includes all sites covered by minimap2 alignments at least 20x coverage, again filling in a score of "0" for sites not output by xPore. **(b)** Similar to a precision recall curve, but with the absolute true positive count rather than the rate (recall) on the x-axis, causing all three missing data filling strategies to output identical curves.

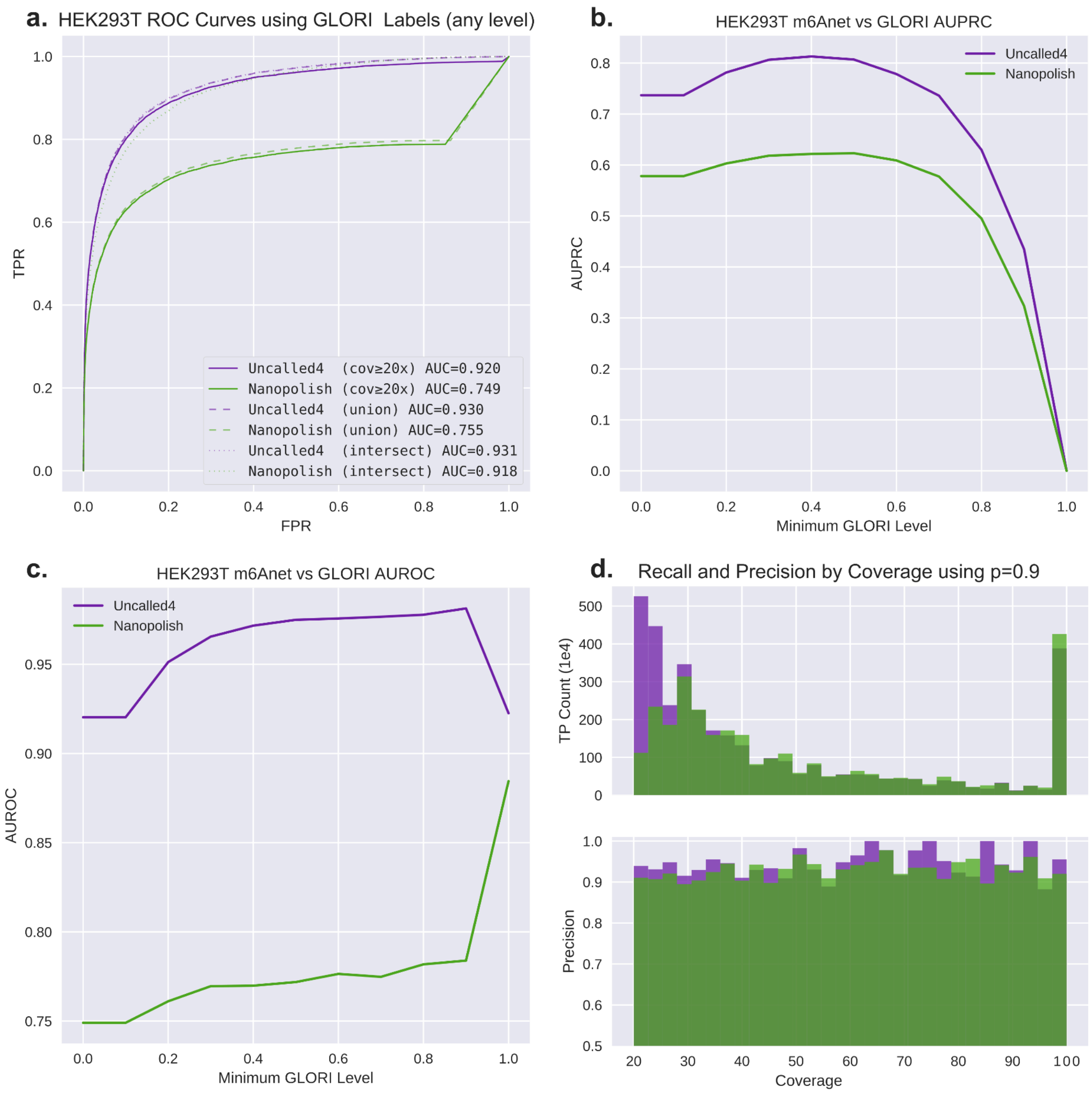

Supplemental Figure 8. Transcript-level m6Anet results on HEK293T WT cell line using Uncalled4 and Nanopolish with GLORI labels. **(a)** ROC curve using either all sites covered by basecalled alignments by at least 20x coverage, the union of all Uncalled4 and Nanopolish output sites, or the intersection of output sites. **(b)** Area under precision-recall curves using different minimum thresholds for the GLORI modification level. Other figures use no threshold on the GLORI level, since the relative performance of Uncalled4 and Nanopolish is similar at all reasonable thresholds. **(c)** Area under ROC curves using different GLORI level thresholds. **(d)** True positive count and precision binned by basecalled read coverage using the recommended m6Anet probability threshold of 0.9. Uncalled4 finds disproportionately more sites at low-coverage, causing the default threshold to overcompensate and reduce recall for high-coverage sites.

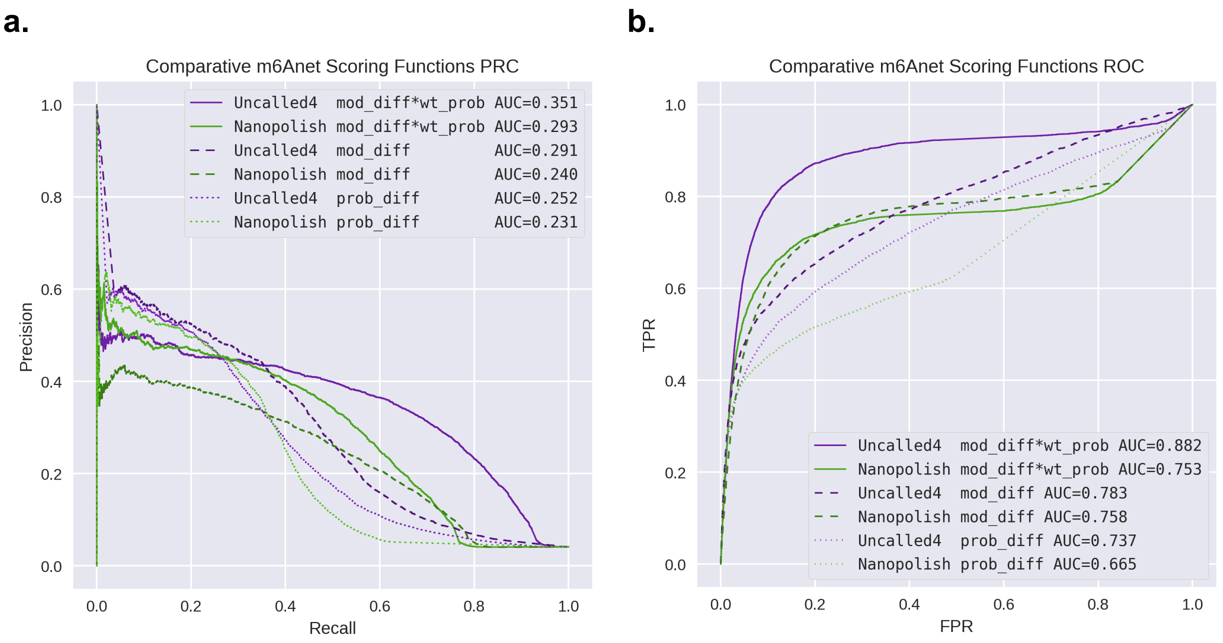

Supplemental Figure 9. (a) Precision-recall and **(b)** ROC curves for different scoring methods for comparative m6Anet modification calling. “mod_diff” refers to the difference in “modification ratio” for the WT and KO sample, which estimates the fraction of reads modified in each sample. “prob_diff” refers to the difference in modification probabilities, which measures the confidence in the modification ratio estimate. Multiplying the WT probability by the difference in modification ratios yields the highest AUPRC for both alignment methods and highest ROC for Uncalled4. Nanopolish ROC is the only case where “mod_diff” performs slightly better.

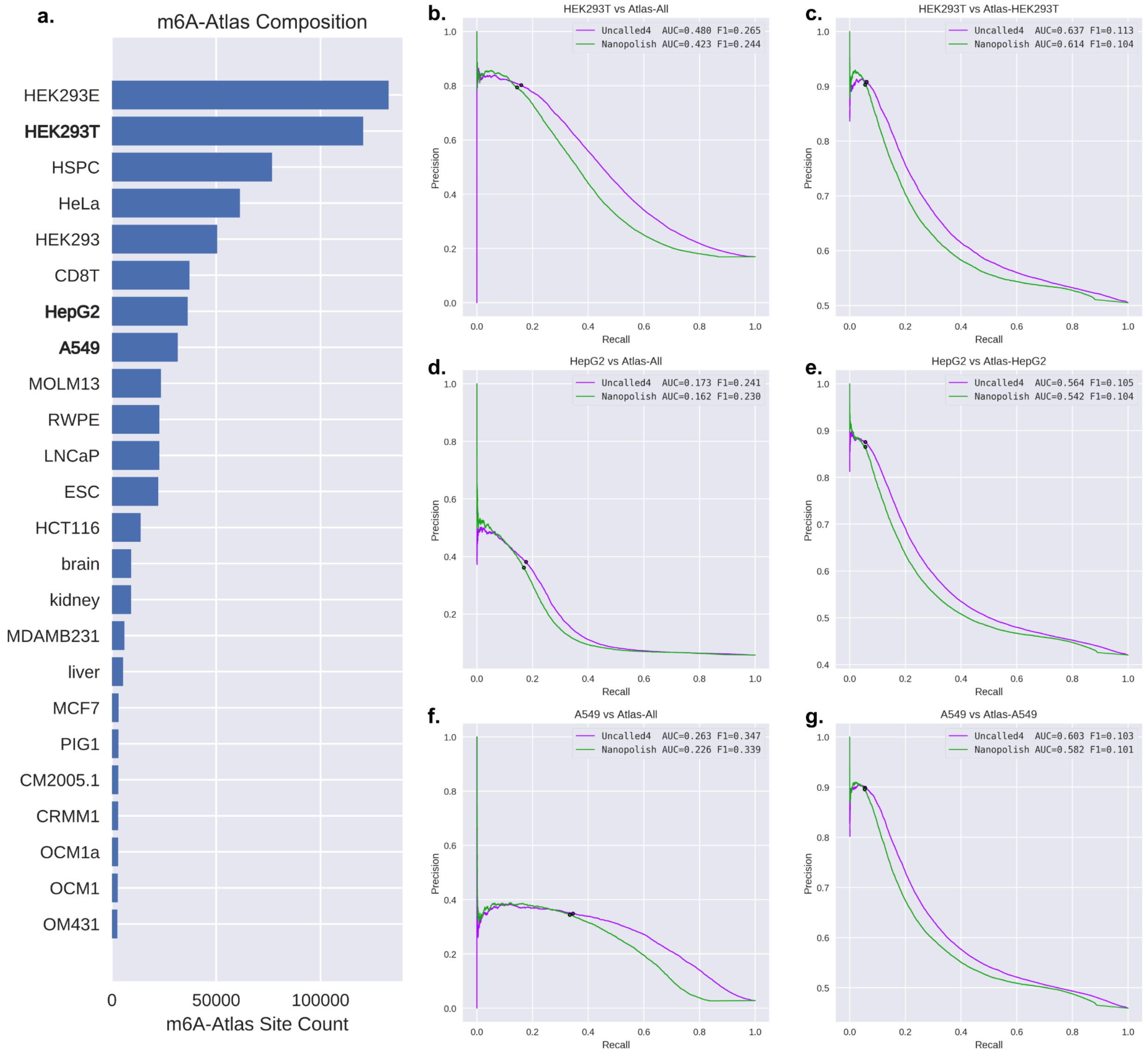

Supplemental Figure 10. Analysis of m6A-Atlas version 2 labels. (**a**) Number of sites from each cell line or tissue in the m6A-Atlas v2. Many sites occur in multiple samples, meaning they are counted twice here. (**b-g**) Precision-recall curves for of m6Anet output using all replicates of HEK293T (**b-c**), HepG2 (**d-e**), and A449 (**f-g**) using m6A-Atlas labels without filtering (**b,d,f**) and filtered for only sites appearing in the matched cell line (**c,e,g**). Colored dots in **b-g** indicate the precision and recall at the recommended probability cutoff of 0.9.

Supplemental Notes

Supplemental Note 1

While RNA004 reportedly has higher accuracy than the previous RNA001 or RNA002 chemistries, we find that reducing the RNA004 model from 9-mers to 5-mers by averaging k-mers that share the same five central bases produces a highly similar model to RNA001 (**Extended Data Figure 1**), suggesting that the pores are highly similar and therefore increased accuracy may be instead due to changes in other aspects of the sequencing chemistry, such as the motor protein or buffer solution. Similarly, we note that RNA001 and RNA002 are highly similar in current means, standard deviation, and dwell time **(Extended Data Figure 1c)**, although the per-run yield and read lengths are generally higher for RNA002.

Uncalled4 can align read signals either directly to the basecalled sequence (signal-to-read alignment, recently introduced in f5c [23]), or *moves* can be translated to reference coordinates based on minimap2 [27] alignments of the basecalled read to generate *ref-moves* to guide signal-to-reference alignment. We apply both methods to *Drosophila melanogaster* r10.4.1 DNA PCR-amplified signal data, and we compare them by measuring the per-k-mer median absolute differences (MAD) between the normalized mean current and the pore model, revealing that signal-to-read alignment yields a slightly lower MAD (0.0998 normalized units) than signal-to-reference alignment (0.1017 normalized units). In contrast, uncorrected *moves* have much higher MAD (*read-moves*=0.4318 and *ref-moves*=0.4164) with an asymmetrical bimodal distribution, compared to an approximately normal distribution centered at zero for both bcDTW methods (**Extended Data Figure 3a)**. We also computed KS statistics using uncorrected *ref-moves* and found it performed worse than KS statistics using any other alignment method (**Extended Data Figure 6**). Signal-to-read alignment avoids reference bias caused by genetic variation between the sample and reference, which could contribute to the reduced MAD value (**Extended Data Figure 3b-c)**. However, such variation will be rare in this dataset, since the genome assembly is of the same *D. melanogaster* Oregon-R strain. Alternatively, a potential downside of signal-to-read alignment is that basecaller errors are generally caused by signal noise, and therefore the reduced MAD may be caused by “over-fitting” the basecalled sequence to the same noisy signal (**Extended Data Figure 3d)**. Furthermore, without additional processing, signal-to-read alignment lacks a shared coordinate system needed to compute a consensus over multiple reads, and no modification detectors have been developed for such alignments. Therefore, while signal-to-read alignment could be a useful reference-free signal analysis method, we limit all subsequent analyses to signal-to-reference alignment.

Supplemental Note 2

Alignment consistency can be directly measured by computing pairwise "distances" between alignment paths: *signal Jaccard distance*, defined as the fraction of raw samples that both methods aligned to the same position, and *signal-to-reference distance*, defined as the average nucleotide distance between reference coordinates over each raw sample for each method (**Fig. 1e**). Uncalled4 is most similar to Nanopolish for r9.4.1 DNA and RNA with a median distance of zero in both metrics (**Table 1**), although higher mean distances indicate outliers from large-scale discordance in certain reads (**Supplemental Figure 2**). f5c is the only other aligner to support r10.4.1 DNA alignment, which also has zero median distance to Uncalled4, but also shows outlier signals. Tombo is more divergent, with fewer than half of all reference coordinates matching Uncalled4 or Nanopolish exactly. It is notable that the distances are generally greater for DNA than RNA, despite the lower error rate in DNA sequencing. This is largely due to intergenic repeats not present in RNA, which are less likely to disrupt Uncalled4 alignments due to the basecaller guided DTW algorithm (**Supplemental Figure 2a**). Large-scale alignment disagreements are also often found near alignment endpoints, near deletions or insertions, or when the molecule speed changes (**Supplemental Figure 2b-d**).

Distance can also be measured between each alignment method and the *ref-moves* used to guide Uncalled4 bcDTW alignment, generally yielding higher distances due to the low-resolution *moves* (**Table 1, Supplemental Table 1**). Uncalled4 consistently has the lowest mean signal-to-reference distance to *ref-moves*, however the median value is lower than Nanopolish/f5c for RNA001 and RNA004, in-part due to more pervasive masking performed by these tools. The difference in mean and median trends likely reflects that Nanopolish/f5c produces more large-scale alignment errors (**Supplemental Figure 2).** Nanopolish also has slightly lower mean and median Jaccard distance in r9.4.1 DNA and RNA, with less than 50% overlap on average for all aligners and less than 2% difference between Uncalled4 and Nanopolish. Despite these small differences, Uncalled4 is the only signal aligner that consistently averages within one nucleotide of the *ref-moves*, indicating consistent accuracy across all sequencing chemistries.

Supplemental Note 3

Uncalled4 iteratively trains pore models by repeatedly aligning signal and averaging signal characteristics for each k-mer (see **Methods**). Beginning with DNA, we trained models to represent canonical nucleotides using PCR-amplified *Drosophila melanogaster* DNA, chosen as its genome contains every possible 11-mer, providing enough context to test different k-mer lengths without requiring more extensive amounts of sequencing. To train models without a prior pore model, we use the *ref-moves* used

to guide our bcDTW algorithm. For r9.4.1 DNA we initialize the process with a single nucleotide (1-mer) model and expand the k-mer length every-other training iteration, alternating which side to append the new nucleotide, to generate a 6-mer model nearly identical to ONT's proprietary model (Pearson's r=0.9995) (**Extended Data Figure 5a**). A similar process was used for r10.4.1, but we initialized with 4-mers and expanded to generate 9-mers, matching ONT's model with slightly lower but strong Pearson's correlation (r=0.9928) (**Extended Data Figure 5b**).

We note a large group of outliers in comparing Uncalled4 and ONT's r10.4.1 models with a consistent NNNNTVTTN motif (N = any base, V = not T). The current for these k-mers is inconsistent with all other k-mers with Ts in two central positions which are otherwise a strong predictor for current level (**Extended Data Figure 5c**). The ONT pore model anomalies are further supported by comparing to ONT's r10.4.1 260bps model, representing a deprecated option for sequencing at slower speed, which has no such outliers and higher similarity to Uncalled4's 400bps model (r=0.9979) (**Extended Data Figure 5d**). Finally, calling 5-methylcytosine CpG methylation (see below) in k-mers containing the TVTT motif yields a weaker modification signal using the ONT model, suggesting poorer alignments in this context (**Supplemental Figure 4**). Taken together, this suggests that the ONT r10.4.1 400bps pore model is inaccurate in the TVTT context, highlighting the value of our fully reproducible pore model training method.

We also trained a r9.4.1 direct RNA (RNA002) model using *in vitro* transcribed (IVT) human HeLa cell line data, using a similar process as r9.4.1 DNA but with additional iterations-per-k-mer-length to adjust for additional noise. We compare with ONT's legacy "rna_r9.4_180mv_70bps" pore model, which is the model used by Nanopolish and Uncalled4 by default for RNA001 and RNA002. The Person's correlation with the ONT model was lower than either DNA model but still strong (r=0.9804). Interestingly, we note a stronger similarity between our RNA002 model and the five central bases of ONT's RNA004 model (r=0.9863), again demonstrating high similarity in current characteristics of these sequencing chemistries (**Supplemental Figure 1b**).

Supplemental Note 4

To compare Uncalled4 and f5c's ability to directly detect 5mCpG methylation, we compared current levels between PCR and 5mCpG D. melanogaster r10.4 data. We included all CpG sites with a 100% modification rate estimated by Guppy and aggregated statistics over a window surrounding each CpG site. We first ran each aligner using f5c's r10.4.1 pore model, which is based on ONT's released model. We performed a two-sample z-test based on the mean and standard deviations of current levels at each position surrounding CpG sites, revealing that 5mCpG generally increases current at the central position, and decreases current at the secondary reader

head and upstream the central position (**Fig. 3b**). We also compute two-sample Komologorov-Smirnov (KS) test statistics to compare current distributions surrounding CpG sites, a nonparametric test which measures distribution similarities regardless of whether current is increased or decreased. Both Uncalled4 and f5c generate two peaks in KS statistics around 5mCpG sites, with a strong peak upstream and a weaker peak downstream, consistent with the double reader head (**Fig. 3b**). Uncalled4 computed a higher primary peak than f5c, indicating a clearer signal of modification, while f5c had a higher secondary peak. We also aligned each dataset using the Uncalled4 pore model for both aligners, increasing KS statistics both at 5mCpG sites and globally (**Supplemental Figure 4c**), likely because the model was trained on the same unmodified D. melanogaster dataset. The difference between KS statistics centered at 5mCpG sites and outside is approximately the same for Uncalled4 using either model, but decreased for f5c since it has not been optimized for the model. As noted previously, the Uncalled4 model performs better in k-mers which contain the TVTT motif, with a stronger primary peak using either aligner (**Supplemental Figure 4d**).

Supplemental Note 5

Presently, the only publicly available ONT BrdU training dataset with signal data was sequenced using legacy r9.5 chemistry, which we find is highly similar to r9.4 signal data (**Supplemental Figure 5a**). We trained two r9.5 6-mer models on reads aligning chromosome 1, an unmodified control model and a BrdU model, using the same model training procedure as r9.4.1. As expected, the resulting BrdU model differs from unmodified DNA most strongly in k-mers with “T” in the central position, and has a strong correlation with the model trained by DNAscent v2 on the same dataset (**Supplemental Figure 5**). To measure the utility of these models, we aligned reads to *S. cerevisiae* chromosome 4 from the unmodified and BrdU datasets (50/50 mixture) using Uncalled4 with either the unmodified control model, the Uncalled4 BrdU model, or the DNAscent BrdU model. We score each k-mer by the absolute current difference between alignments using either BrdU model or the control model, only considering k-mers with either one “T” at the central position (BrdU-positive in the BrdU dataset) or no “T”s (always BrdU-negative), yielding a higher area under the receiver operating curve (AUROC) and the precision-recall curve (AUPRC) when the Uncalled4 BrdU model is used (**Fig. 3d**, **Supplemental Figure 5c**).

Supplemental Note 6

While transcript-level modification calling is a unique strength of ONT direct RNA sequencing, gene-level modification calls may be more robust by combining information from multiple isoforms, especially when it is ambiguous from which transcript the read originated. Uncalled4 can perform genome alignment given spliced basecaller alignments, unlike Nanopolish and Tombo which only support transcriptome alignment

for RNA. An alternative to direct genome alignment is mapping transcript-level calls to genome coordinates and averaging the probabilities ("t2g", **Extended Data Figure 7a**), a process that is built-in to xPore. While we only include primary alignments when computing transcript-level modification calls, we include all multi-mapping reads for *t2g* mapping with Uncalled4 and Nanopolish to preserve ambiguously aligning reads from multi-isoform transcripts, finding that this increases AUPRC and AUROC compared to primary alignments (**Fig. 4a, Extended Data Figure 7**). Note that this would not be appropriate for transcript-level analysis since the read aligner may clip alternatively spliced regions, and only primary alignments are included for Tombo as each read is fundamentally associated with a single alignment in its FAST5 format. Uncalled4 spliced genome alignment outperforms KS statistics or xPore with *t2g* mapping, indicating some information is lost in transcriptome alignment even when all multi-mapping reads are included (**Extended Data Figure 8**). A likely source of reduced performance for *t2g* mapping is that coverage for alternatively spliced genes may be split between multiple isoforms, so the initial modification scores will be based on the lower-coverage transcriptome alignments prior to gene-level averaging. Spliced genome alignments cannot be input to other methods like xPore or m6Anet, as they cannot handle splicing or large chromosomes.

Supplemental Note 7

m6Anet outputs probabilities that each candidate site is modified, and the choice of threshold determines the precision and recall. Using the recommended probability cutoff of 0.9 in a single HEK293T replicate, Uncalled4 has higher recall (27% vs 23%) and precision (94% vs 92%) than Nanopolish based on GLORI labels. Uncalled4 finds disproportionately more true positives (TPs) at low-coverage sites (20-30x) than Nanopolish, but fewer TPs at high-coverage sites (>30x, **Supplemental Figure 8d**), likely because m6Anet training scales the probabilities to compensate for the larger fraction of low-coverage sites in Uncalled4 alignments. We therefore reduced the modification probability threshold for each aligner such that both have 90% overall precision based on GLORI-seq labels (threshold=0.81 for Uncalled4, 0.88 for Nanopolish), yielding consistently higher recall for Uncalled4 (39% vs 26% overall) with similar precision across all coverage levels (**Fig. 4c**). To further validate m6Anet precision, we measure the false positive (FP) rate of m6Anet using Uncalled4 or Nanopolish on *in vitro* transcribed HeLa RNA002 data containing no modifications, yielding a FP rate of 0.031% for Uncalled4 (37 FP sites) compared to 0.134% for nanopolish (143 FP sites) using the recommended 0.9 probably threshold, despite Uncalled4 outputting 11% more sites overall than Nanopolish.

To compare m6Anet with comparative methods, we used the difference in modification rate at each site between the WT and METTL3 KO samples multiplied by the WT modification probability as the differential modification score, which yielded higher

AUPRC scores than scoring based on modification rate or probability differences alone (**Supplemental Figure 9**). Uncalled4+m6Anet has higher AUPRC and AUROC than all other comparative methods. (**Fig. 4a, Extended Data Figure 6**). Interestingly, while DRACH-context KS statistics perform worse than m6Anet using the same alignment method, Nanopolish+m6Anet has a lower AUROC and AUPRC than Uncalled4+KS statistics, demonstrating that the alignment method has a larger impact than the downstream detection method in this context.

Supplemental Note 8

The m6A-Atlas has uneven representation across cell lines, most notably in HEK293T with over 100,000 gene-level sites, while others have no representation (NA12878, HMEC, K562) (**Supplemental Figure 10a**). However, we find that the relative AUPRC and AUROC scores for Uncalled4 and Nanopolish are similar for HEK293T when using GLORI labels, the full m6A-Atlas v2, or HEK293T-specific m6A-Atlas sites (**Fig. 4b**, **Supplemental Figure 10b-c**). Similar relative performance is also observed using the full atlas or cell-specific labels for HepG2 and A549, suggesting the full m6A-Atlas may be used as a proxy for precision in a variety of cell lines (**Supplemental Figure 10c-d)**. We note that the putative "recall" is underestimated when combining labels from many samples, since we do not expect consistent modification under all conditions. For all analyses that include multiple cell lines, we call each positive modification site a "putative true positive" (pTP) if it occurs in any sample in the m6A-Atlas, and accordingly measure "putative positive predictive value" (pPPV) instead of PPV/precision, acknowledging that this is an imperfect truth set and accuracy will vary between cell lines.
