# Supplementary material for: Uncalled4 improves nanopore DNA and RNA modification detection via fast and accurate signal alignment
Source: Nat Methods. 2025 Mar 28;22(4):681–91. doi: 10.1038/s41592-025-02631-4 (PMC11978507; doi:10.1038/s41592-025-02631-4)
Supplement: Supplementary file 2 — Reporting Summary [file 41592_2025_2631_MOESM2_ESM.pdf]

Corresponding author(s): Sam Kovaka
Last updated by author(s): Nov 14 2024

# Reporting Summary

Nature Portfolio wishes to improve the reproducibility of the work that we publish. This form provides structure for consistency and transparency in reporting. For further information on Nature Portfolio policies, see our Editorial Policies and the Editorial Policy Checklist.

## Statistics

For all statistical analyses, confirm that the following items are present in the figure legend, table legend, main text, or Methods section.

| n/a | Confirmed | |
|---|---|---|
| ☐ | ☒ | The exact sample size (*n*) for each experimental group/condition, given as a discrete number and unit of measurement |
| ☒ | ☐ | A statement on whether measurements were taken from distinct samples or whether the same sample was measured repeatedly |
| ☐ | ☒ | The statistical test(s) used AND whether they are one- or two-sided<br>*Only common tests should be described solely by name; describe more complex techniques in the Methods section.* |
| ☒ | ☐ | A description of all covariates tested |
| ☒ | ☐ | A description of any assumptions or corrections, such as tests of normality and adjustment for multiple comparisons |
| ☐ | ☒ | A full description of the statistical parameters including central tendency (e.g. means) or other basic estimates (e.g. regression coefficient) AND variation (e.g. standard deviation) or associated estimates of uncertainty (e.g. confidence intervals) |
| ☒ | ☐ | For null hypothesis testing, the test statistic (e.g. *F*, *t*, *r*) with confidence intervals, effect sizes, degrees of freedom and *P* value noted<br>*Give P values as exact values whenever suitable.* |
| ☒ | ☐ | For Bayesian analysis, information on the choice of priors and Markov chain Monte Carlo settings |
| ☒ | ☐ | For hierarchical and complex designs, identification of the appropriate level for tests and full reporting of outcomes |
| ☐ | ☒ | Estimates of effect sizes (e.g. Cohen's *d*, Pearson's *r*), indicating how they were calculated |

*Our web collection on statistics for biologists contains articles on many of the points above.*

## Software and code

Policy information about availability of computer code

| | |
|---|---|
| Data collection | Sequencing was performed using Oxford Nanopore MinKNOW v5.2.8 |
| Data analysis | Basecalled using Guppy v6.4.8 for most data, except for Tombo where v4.0.1 was used. Basecalled alignments using minimap2 v2.16. Signal alignments using Uncalled4 v4.0.0, Nanopolish v0.13.3, Tombo v1.5.1, and f5c v1.3. |

For manuscripts utilizing custom algorithms or software that are central to the research but not yet described in published literature, software must be made available to editors and reviewers. We strongly encourage code deposition in a community repository (e.g. GitHub). See the Nature Portfolio guidelines for submitting code & software for further information.

## Data

Policy information about availability of data

All manuscripts must include a data availability statement. This statement should provide the following information, where applicable:

- Accession codes, unique identifiers, or web links for publicly available datasets
- A description of any restrictions on data availability
- For clinical datasets or third party data, please ensure that the statement adheres to our policy

Drosphila sequencing data generated for this study is available at SRA bioproject PRJNA634693. All other datasets were obtained from publicly available sources. The E. coli rRNA data was obtained from [29] (SRA bioproject PRJNA634693). The constitutively incorporated BrdU dataset and matched control were from [30] (SRR8991355 and SRR8991351). The IVT HeLa cell direct RNA sequencing data used to train the RNA002 model was obtained from [53] (SRR23950400). HEK293T

wildtype and METTL3 knockouts was obtained from [31] (PRJEB40872). NA12878 data was obtained from [54] (https://github.com/nanopore-wgs-consortium/NA12878). HMEC data was obtained from [55] (GEO accession GSE132971). All other human cell line data is from the Singapore Nanopore Expression Project (PRJEB40872)

## Human research participants

Policy information about studies involving human research participants and Sex and Gender in Research.

| | |
|---|---|
| Reporting on sex and gender | N/A |
| Population characteristics | N/A |
| Recruitment | N/A |
| Ethics oversight | N/A |

Note that full information on the approval of the study protocol must also be provided in the manuscript.

# Field-specific reporting

Please select the one below that is the best fit for your research. If you are not sure, read the appropriate sections before making your selection.

☒ Life sciences ☐ Behavioural & social sciences ☐ Ecological, evolutionary & environmental sciences

For a reference copy of the document with all sections, see nature.com/documents/nr-reporting-summary-flat.pdf

# Life sciences study design

All studies must disclose on these points even when the disclosure is negative.

| | |
|---|---|
| Sample size | Drosophila genomes were sequenced as this genome contains very possible 11-mer, allowing sufficient context to produce 9-mer k-mer models. |
| Data exclusions | We excluded Drosophila r10.4.1 260bps sequencing chemistry as this sequencing method was deprecated by ONT |
| Replication | All experiments were computational and can be replicated by running the command again |
| Randomization | Not relevant, no experimental groups required |
| Blinding | Not relevant, no experimental groups required |

# Reporting for specific materials, systems and methods

We require information from authors about some types of materials, experimental systems and methods used in many studies. Here, indicate whether each material, system or method listed is relevant to your study. If you are not sure if a list item applies to your research, read the appropriate section before selecting a response.

## Materials & experimental systems

| n/a | Involved in the study | |
|---|---|---|
| ☒ | ☐ | Antibodies |
| ☒ | ☐ | Eukaryotic cell lines |
| ☒ | ☐ | Palaeontology and archaeology |
| ☐ | ☒ | Animals and other organisms |
| ☒ | ☐ | Clinical data |
| ☒ | ☐ | Dual use research of concern |

## Methods

| n/a | Involved in the study | |
|---|---|---|
| ☒ | ☐ | ChIP-seq |
| ☒ | ☐ | Flow cytometry |
| ☒ | ☐ | MRI-based neuroimaging |

## Animals and other research organisms

Policy information about studies involving animals; ARRIVE guidelines recommended for reporting animal research, and Sex and Gender in Research

| | |
|---|---|
| Laboratory animals | 15 newly eclosed (one day old) D. melanogaster males of the Oregon R strain (Bloomington stock center, #5) |
| Wild animals | Did not involve wild animals |
| Reporting on sex | Sex was not relevant to the study, but all individuals were male according to the stock facility |
| Field-collected samples | Did not involve field-collected samples |
| Ethics oversight | Not required for D. melanogaster |

Note that full information on the approval of the study protocol must also be provided in the manuscript.
